# Prevalence and determinants of women’s satisfaction on the quality of safe abortion service in Northwest Ethiopia

**DOI:** 10.1186/s13690-022-00897-0

**Published:** 2022-05-26

**Authors:** Kiros Terefe Gashaye, Asefa Adimasu Taddese, Tilahun Yemanu Birhan

**Affiliations:** 1 Department of Obstetrics and Gynecology, School of Medicine, College of Medicine and Health Sciences, University of Gondar, Gondar, Ethiopia; 2 Department of Epidemiology and Biostatistics, Institut of Public Health, College of Medicine and Health Science, University of Gondar, Gondar, Ethiopia

**Keywords:** Safe abortion, Post-abortion contraception, Women, Quality, Ethiopia

## Abstract

**Background:**

The standard of treatment in developing countries is harmed by a complex political landscape, an uncertain economic climate, rapid population growth, and clients are constantly complaining about the poor health system. Patients’ assessments of the benefits and shortcomings of the service provided to them are expressed as satisfied or dissatisfied. The objective of this study was to determine the magnitude of women’s satisfaction on quality of safe abortion and factors associated with it in Northwest Ethiopia.

**Methods:**

Institution-based cross-sectional study design was done to collect data from 618 women in the selected health facilities in Northwest Ethiopia. Women having the gestational trophoblastic disease (partial mole) and those who cannot hear or are seriously ill during the data collection period were excluded. The study subjects were chosen using a randomization process, and each participant was questioned after receiving all necessary abortion treatment and giving verbal consent. Both bivariable and multivariable logistic regression analysis was carried out to determine covariates significantly associated with women’s satisfaction on quality of abortion.

**Results:**

The highest proportion of women who were reasoned out to terminate the fetus in the current pregnancy was due to financial problems (29.36%) and partner coercion (23.85%). Client satisfaction with safe abortion services in the study region was 25.10% (95% CI; 21.81–28.70). Women were 53.2% satisfied with the art of treatment/interpersonal abilities, 59.2% satisfied with the professional quality of care professionals, 54.5% satisfied with the physical environment, and 49.8% satisfied with the structure of the health care system, respectively. In the multivariable logistic regression analysis, women’s living solely 0.47(0.26–0.87) & living with 2 to 4 people 0.11(0.04–0.29), college and above level of education 1.78(1.01–3.15), wanted the status of pregnancy 0.44(0.23–0.85) and post-abortion contraceptive users 1.70(1.01–2.89) were factors significantly associated with women’s satisfaction level.

**Conclusion:**

In this study, one-fourth of the women were satisfied with the quality of safe abortion services. Family size, women’s educational status, maternity status, and post-abortion contraception use were predictors of women’s satisfaction with the quality of abortion. Ethiopia’s government should concentrate on addressing contraception needs in order to prevent women from having multiple abortions.

**Supplementary Information:**

The online version contains supplementary material available at 10.1186/s13690-022-00897-0.

## Plain english summary

The quality of safe abortion practice assists an individual in avoiding undesired pregnancy and achieving their intended child within their planning time frame, and viable means of preventing repeated abortion should be provided to women with a variety of options. Induced abortion remains an alarming global public health concern due to low quality health services and lesser technical capabilities of health care providers in the study area, despite the restricted availability of quality safe abortion practices and the well-deserved satisfaction of women. As a result, the purpose of this study was to determine women’s satisfaction with the quality of abortion, which is not widely addressed in the research domain. All health institutions which provide legal abortion service in the study area were included. The sample size was proportionally alocated in each health facility based on the previous patient flow, and the study participants were rigorously selected.

The proportion of women’s satisfaction on the quality of safe abortion practice was 25% which is low as compared to other studies conducted in Ethiopia. Women who live solely, living with multiple family, having highest education status, and using post-abortion contraceptive were factors associated with women’s satisfaction on the quality of safe abortion practice. The intervention should concentrate on addressing contraception needs in order to prevent women from having multiple abortions.

## Background

Despite decades of experimentation, most Sub-Saharan African countries’ health systems are underperforming in terms of low funding, organizational and management inefficiency, poor quality of health services, inequities in health workforce distribution, and low capacity for planning, budgeting, and good governance [1]. Contextual factors like a complex political landscape, an uncertain economic climate, and rapid population growth thwart efforts to improve the efficiency of these health systems. Patients are dissatisfied due to a weak health system, which other disease pressures, causes patient dissatisfaction [2]. Patients’ dissatisfaction with medical care may play a role in unsafe abortions, as well as increased maternal mortality and morbidity [3–6]. Around 46 million women have induced abortions each year around the world, with 78% of them living in developing countries. However, as figures from countries with legal, safe abortion or a successful harm reduction model show, maternal mortality from abortion can be avoided [7, 8]. Furthermore, client service-related satisfaction levels are critical for assessing the institution’s health-care quality and identifying factors that influence clinical performance, patient retention, and medical malpractice [9–14], and to see whether the service meets the clients’ needs, such as having a choice of services, receiving reliable and complete information, receiving technically competent treatment, having good interactions with providers, having the quality of care, and a constellation of relevant services [5].

As a result, several studies in developing countries have discovered that inability to support oneself and family, having too many children, and being young and in school are all determinant factors for safe abortion treatment [15–20]. Furthermore, research conducted in several countries revealed that hospital post-abortion treatment is still limited due to financial constraints, scarcity of knowledge of post-abortion facilities, lack of accurate information, stigma and sexism, religious conviction, male dominance, and family or community pressure [21–24]. Specifically, for client satisfaction levels many factories were listed like the educational status, occupational status, laboratory prescription, the opportunity given to take part in decisions, equity of treatment, advice given by service providers, availability of service providers, and availability of drugs (anti-pain) and toilet access as predictors of clients satisfaction level in the previous studies [25–27].

On the other hand, low healthcare quality and dissatisfied clients in the healthcare system are ongoing issues, especially in developing countries [28]. Client satisfaction with health services is clinically important because happy patients are more likely to adhere to medication, participate actively in their care, continue to use medical care services, and refer others to the center [29]. A satisfied patient will refer four or five people to the center, whereas an unhappy patient will complain to twenty or more people [30]. Because there have been no studies on patient satisfaction in our settings, the goal of this study was to find out how common patient satisfaction is among women who have had abortions. The findings of this study serve as a model for future research and serve as baseline data for policymakers and authorized bodies in our setting.

## Methods and materials

### Study design and setting

An institution-based cross-sectional study design was done to collect data from 618 women in the selected private and public health facilities in northwest Ethiopia. According to the Ethiopian Central Statistics Agency, the region has a projected population of 21.5 million people, about 80% of whom are rural farmers [31]. The region has 80 hospitals (5 referral, 2 general, and 73 primaries), 847 health centers, and 3342 health posts [32, 33].

### Study populations and sampling procedure

All women who are attending abortion services during the study period in the selected hospitals of the region are the source population for the study. Women excluded from the study are those having the gestational trophoblastic disease (partial mole) and those who cannot hear or are seriously ill with a coma during the data collection period. The sample for each hospital was arranged based on their patient flow by reviewing the 6-month report of the previous year. After proportional allocation of the samples for each health institution, Randomization was performed to select the study subjects.

### Data collection method

and a participant was interviewed based on their exit after they received all the necessary abortion care and after informed verbal consent has been taken.

An assessment tool was adapted from previously reported literature [25, 34]. The satisfaction level of women’s was measured by 26 five-point Likert Scale questions which range between 0 and 4; scale(0==neutral,1 = Strongly disagree, 2 = disagree, 3 = Agree & 4 = Strongly agree) the scores for each domain were calculated by summing the answers to all items in each domain: The overall and component-wise satisfaction was classified into two categories satisfied and dissatisfied by using cut of point calculated using the demarcation threshold formula: {(total highest score-total lowest score)/2} + Total lowest score [35, 36]**.**

### Data management and analysis

The responses of the study subjects were coded and entered in to Epi-info 4.6 and then exported to Stata 14 for analysis. Descriptive statistics and summary measures of the variables were conducted to see the characteristics of the study subjects. The crude and adjusted odds ratio (OR) with 95% confidence interval were carried out from Bi-variable and Multi-variable logistic regression analysis in order to measre the strength of association between the response and the explanatory variables. Before preeding to the multivariable logistic regression, variables which had *p*-value < 0.2 in the bi-variable logistic regression were included in the mulutivariable logistic regression. Finally, p-value < 0.05 was considered statistically significant for all explanatory variables at multivariable logistic regression.

To see the the model fitness, Hosmer-Lemeshow Goodness of Fit test and Multicolliniarity were checked to minimize bias. The final model’s goodness of fit was assessed using the Hosmer–Lemeshow Goodness of Fit Test, with *p*-values greater than 0.05 considered as the model’s fit to the logistic regression. We use the Variance Inflator Factor (VIF) to investigate the instability of the effect size of predictors as a result of high collinearity among themselves. The mean, variance inflation factors (VIF) cutoff point of 10 was used to test for multicollinearity.

## Results

### The socio-demographic characteristics of the respondents

About six hundred eighteen women have participated in this study. Of them, 53.1% were found between18–23 age groups (median age 23 ± 4.6 years). More than half (65.9%) of them were getting service in the selected private and government hospitals and the remaining were in the health center. When we are looking at the distribution of samples based on the types of health facilities, 374 governmental, 44 NGO, and 200 private health institutions. The number of urban resident women was about 87.5 and 66.2% of them were living together with 2–4 family members. Out of the respondents, the maximum number, 233(37.7%) of women were getting pregnant without a voluntary relationship (never married) in this study. Eighty-seven (22.5%) of respondents’ partners have their first degree and above, while 120 (26.3%) are self-employee. The respondent’s monthly income was ranged from 100 and 18, 000 Ethiopian Birr, with a mean of 2845.7 Ethiopian Birr (Table 1).

**Table 1:** The socio-demographic characteristics of study participants in the selected area of northwest Ethiopia, 2020

Variable Name	Category	Frequency (%)
Women age category	13–18	26 (4.2)
	18–24	328 (53.1)
	24–28	174 (28.2)
	28–34	63 (10.2)
	> 34	27 (4.4)
Residence (*N* = 618)	Urban	541(87.5)
	Rural	77 (12.5)
Number of peoples living together	1	100 (16.2)
	2 to 4	409 (66.2)
	≥ 5	109(17.6)
Level of health institutions(N = 618)	Health Center	211(34.1)
	Hospital	407(65.9)
Towns where health Facilities are located (*N* = 618)	Addis-Zemen	40 (6.5)
	Bahirdar	300 (48.5)
	Debark	25 (4.0)
	Debre-tabor	15 (2.4)
	Gondar	201 (32.5)
	Woreta	37 (6.1)
Husband/Partner occupational Status (Total = 456)	Student	106(23.2)
	Government Employe	262(57.4)
	Farmer	41 (8.9)
	Daily laborer	47 (10.3)
Average daily family income	≤ 1500 ETB	278(45.0)
	1501–3000 ETB	157 (25.4)
	≥ 3001 ETB	183(29.6)
Marital Status (Total = 618)	Never married	233 (37.7)
	Currently married	100 (16.2)
	Separated	154(24.9)
	Having boyfriend	131 (21.2)
Types of health facility (*N* = 618)	Government	374 (60.5)
	NGO	44 (7.1)
	Private	200(32.4)
Women occupational status (Total = 613)	Student	238(38.5)
	Government Employe	143 (23.1)
	Housewife	94 (15.2)
	Housemaid	20(3.2)
	daily labor	69(11.2)
	Commercial sex worker	54(8.7)
Husband/Partner Educational Level (Total = 362)	Informal education	116(32.0)
	Primary & secondary	158(43.7)
	Collage and above	88(24.3)
Women educational Level (Total = 606)	Informal Education	167(27.0)
	Primary & secondary	211 (34.1)
	Collage and above	240(38.8)

### The medical and reproductive history of women

Of the 178 participants who were asked about their age at first marriage, about 115 (65%) of them were married after the age of eighteen years while 63 (35%) were before reaching eighteen years. Even though, the majority (57%) of them were first-time pregnant when aged between 18 to 22 years, around 53 (9%) of women were pregnant at the age of less than 18 years. Four hundred sixteen (67%) participants were primipara and 202 (33%) were multiparous while 434 (70%) are nulliparous. In this study only, 53 (9%) had a history of abortion, of which 28 (53%) had spontaneous and 25 (47%) had induced abortion. One hundred seven safe abortions were wanted pregnancies.

Of all the cases that underwent safe abortion service during the data collection period, 544 (88%) were in the first trimester and 74 (12%) in the second trimester. Forty-three cases (7%) faced safe abortion-related complications of which 17 (2.8%) cases experienced severe lower abdominal cramp, 15 (2.4%) cases had massive vaginal bleeding and 11 (1.8%) experienced signs of infection. From the interventions done to manage safe abortion-related complications, 17 (2.8%) had a blood transfusion and 5 (1%) of cases were given antibiotics and analgesics. Nothing was done to a significant proportion of 21 (3.4%) of the case. About linkage to other reproductive health issues, 209 (34%) of the cases did not know their HIV/AIDS and other sexually transmitted infections including syphilitic screening (VDRL) status and only 41 (6.6%) of the cases had cervical cancer screening. Of all the cases that underwent safe termination, 55 (9%) cases have a plan to be pregnant soon, of which 20 (40%) planned to be pregnant again between six months and one year (Table 2).

**Table 2:** The reproductive characteristics of study participants in the selected health institutions in Northwest Ethiopia, 2020

Variables	Category	Frequency (%)
Age at first marriage (*N* = 178)	< 18 years	63 (35.4)
	≥18 years	115 (64.6)
Age of women at first pregnancy (*N* = 599)	13–18 year	53 (8.85)
	18–23 year	344 (57.43)
	23–27 year	188 (31.39)
	≥ 28 year	14 (2.34)
Number of pregnancy (Gravidity)(*n* = 618)	1	416 (67.3)
	2–4	173 (28.0)
	≥5	29 (4.7)
Previous gestational age	First trimester	45 (83.3)
	Second trimester	9 (16.7)
wanted status of current pregnancy	Wanted	107(17.3)
	Unwanted	511(82.7)
**Linkage to other Reproductive health issue**		
HIV/AIDS screening	Yes	409 (66.2)
	No	178 (28.8)
	I don’t know	31(5.0)
Cervical cancer screening	Yes	41(6.6)
	No	440 (71.2)
	I don’t know	137 (22.2)
STI screening	Yes	409(66.2)
	No	129 (20.9)
	I don’t know	80 (12.9)
History of abortion	Yes	53 (8.6)
	No	565 (91.4)
Types of previous abortion	Spontaneous	28 (52.8)
	Induced	25 (47.2)
Number of delivery (Live birth +stillbirth)	0	434(70.2)
	1	95 15.4)
	2–4	73 11.8)
	≥5	16 (2.6)
Abortion complication	Yes	43 (7.10)
	No	563 (92.90)
Current gestational age	1st trimester	544 (88.0)
	2nd trimester	74 (12.0)
Type of complication	Vaginal bleeding	15(34.8)
	Infection/offensive odor	11(25.6)
	Abdominal cramping and pain	17 (39.5)
Interventions done	Blood transfusion	17 (39.5)
	Anti-pain and antibiotic	5 (11.6)
	Nothing	21 (48.8)
Desire to pregnancy soon	Yes	55 (8.9)
	No	563 (91.1)
Times of future pregnancy	< 6 months	12 (24.0)
	6–1 year	20 (40.0)
	≥ 1 year	18 (36.0)

### Reasons to terminate a wanted pregnancy

**Fig. 1:**
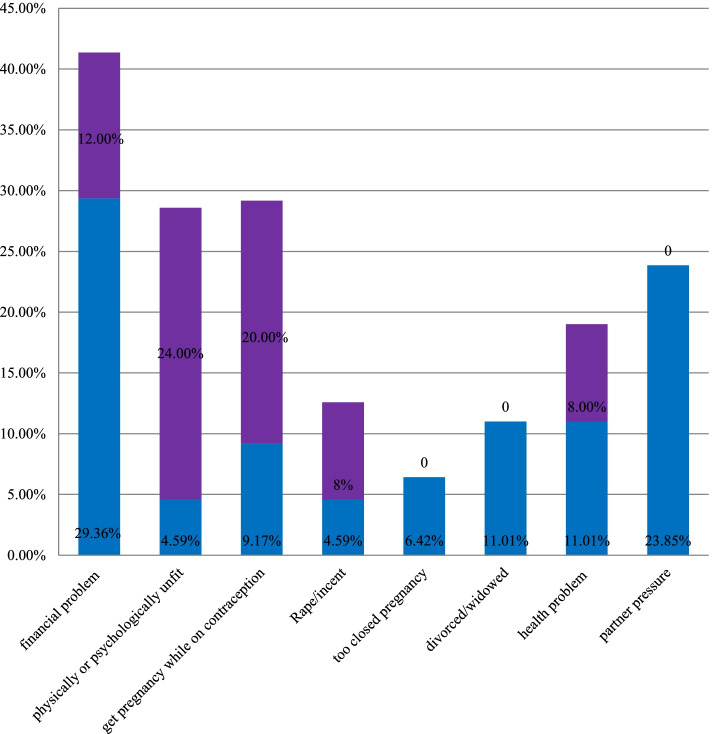
Reasons for the previous abortion among study participants

Financial issues (29.36%) and social pressure (23.85%) are the most common reasons for women to terminate their pregnancies in the current pregnancy. Previously, 24% of women had abortions due to being physically or psychologically unfit, 20% due to contraceptive failure, 8% pregnancy due to abuse, 8% due to health-related problems, and 12% due to financial problems, while 28% of women had abortions due to being physically or psychologically unfit, 20% due to contraception failure, 8% pregnancy due to rape, 8% due to health-related problems, 8% due to financial problems, and 28% of clients did not suggest any reason (Fig. 1).

### Procedures are done for previous and current abortion

**Fig. 2:**
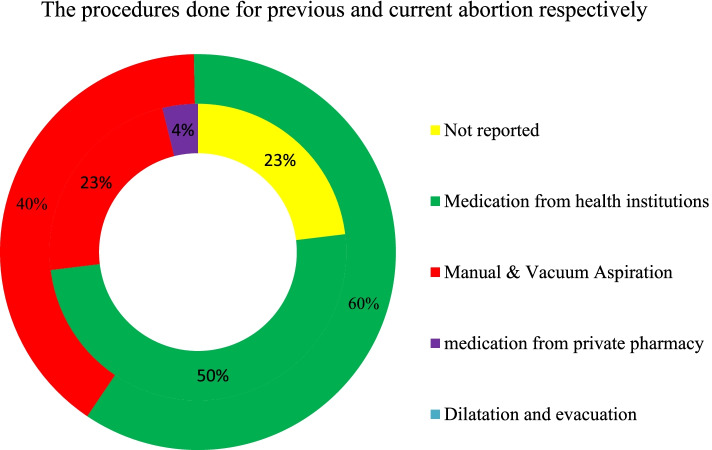
The percentage distribution of methods of uterine evacuation for their previous and current abortion in northwest Ethiopia, 2020

In previous abortion procedures, 50% of the women said they were given prescriptions at health institutions; manual vacuum aspiration was used in 23% of the cases at health institutions; 3.8% took medications from private pharmacies, and 23% did not disclose the methods that were used. However, 368 (60%) of the current abortions were performed with medication, 249 (40%) with manual vacuum aspiration, and 2 with dilatation and evacuation (Fig. 2).

### The service satisfaction level of women

**Fig. 3:**
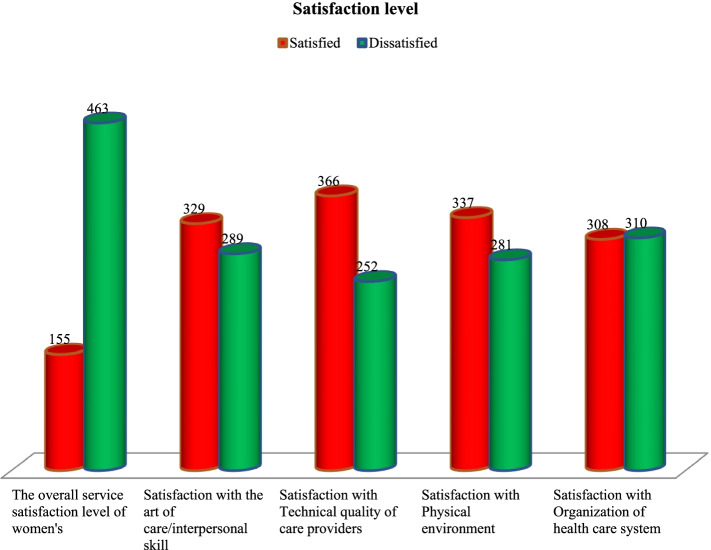
The service satisfaction levels of women’s in the selected private and public hospitals in the Northwest Ethiopia, 2020

**Fig. 4:**
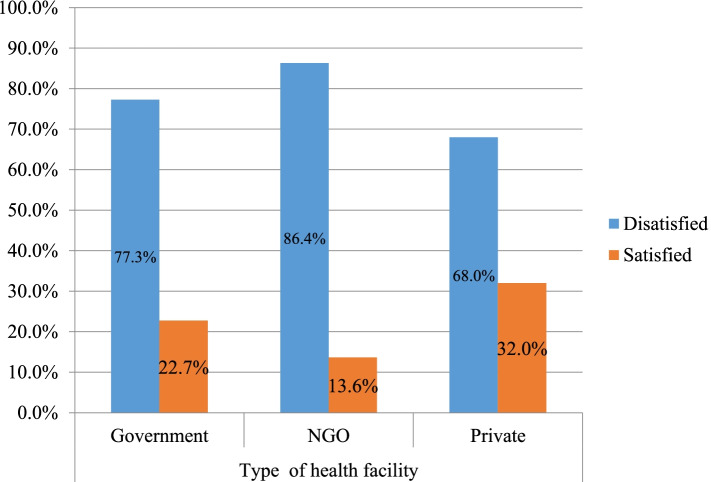
The satisfaction level of women’s based on the types of health institutions in Northwest Ethiopia, 2020

Client satisfaction with safe abortion services in the study region was 25.08%, with a 95% CI (21.81–28.70). Women were 53.2% satisfied with the art of treatment/interpersonal abilities, 59.2% satisfied with the professional quality of care, 54.5% satisfied with the physical environment, and 49.8% satisfied with the structure of the health care system. In this analysis, women’s satisfaction with the professional standard of treatment accounted for the highest proportion (Fig. 3). Women were satisfied in 32.0, 22.7, and 13.6% of private, government, and non-profit health institutions, respectively. Clients in private health facilities were happier than those in government and non-profit health facilities (Fig. 4).

### Factors associated with women’s satisfaction with the quality of abortion at Northwest Ethiopia

In the multivariable logistic regression analysis, variables such as women’s living solely (AOR = 0.47; 95% CI, 0.26–0.87) & living with 2 to 4 people (AOR = 0.11; 95% CI, 0.04–0.29), college and above level of education (AOR = 1.78; 95% CI, 1.01–3.15), wanted pregnancy (AOR = 0.44; 95% CI, 0.23–0.85) and post-abortion contraceptive (AOR = 1.70, 1.01–2.89) were factors significantly associated with women’s satisfaction with abortion (Table 3).

**Table 3:** Factors associated with women’s satisfaction on quality of safe abortion practice in Northwest Ethiopia, 2020

Variables	Categories	COR [95% CI]	AOR [95% CI]
Women educational status	No formal education	0.63(0.38–1.04)	1.22(0.62–2.38)
	Primary & secondary education	1	1
	Collage & above	1.09(0.72–1.66)	1.78(1.01–3.15)*
Partner occupational status	Student	1.35(0.55–3.29)	1.13(0.43–2.98)
	Employed in Gov’t & private institution	1.74(0.77–3.91)	1.64(0.68–3.91)
	Farmer	0.68(0.20–2.26)	0.84(0.24–2.94)
	Daily labor	1	1
Family size living together	Single	1	1
	2–4 people	0.59(0.37–0.94)*	0.47(0.26–0.87)**
	≥5 people	0.13(0.05–0.29)**	0.11(0.04–0.29)**
Information about abortion	Yes	0.65(0.41–1.05)	0.67(0.35–1.31)
	No	1	1
wanted status of pregnancy	Yes	0.49(0.29–0.86)**	0.44(0.23–0.85)**
	No	1	1
Post-abortion contraceptive used	Yes	2.13(1.42–3.19)**	1.70(1.01–2.89)*
	No	1	1

^*^Significant at alpha 0.1

^**^Significant at alpha 0.05

^***^Significant at alpha 0.01

## Discussion

Various studies reported that abortion increases the risk of complications, such as placenta previa, which increases the risk of fetal malformation, perinatal death, and excessive bleeding during labor. However, increasing access to safe abortion services is the most effective way of reducing the burden of unsafe abortion, which is accomplished by expanding safe pregnancy termination options. In this study, the satisfaction levels of women with medical abortion were examined. As a result, 25% of the women were satisfied with the quality safe abortion service, lower than studies conducted in Tigray and Jimma [25, 34]. This disparity could be attributed to a difference in attitude and skill among health care workers, an inability to implement guidelines, and a lack of updated instruments at the health institution. Furthermore, limited access to safe abortion facilities may be a barrier to universal access to reproductive health care; as a result, Ethiopians oppose abortion and face widespread prejudice.

In this study, we found that covariates significantly associated with women’s satisfaction on quality of safe abortion, among those women who have college and above educational level 1.78 times more likely satisfied on quality of safe abortion than non-educated women consistent with studies conducted in Tigray and Jimma [25, 34]. This is because educated women have a better understanding and attitude about the risks and benefits of abortion in their lives; as a result, educated women are more likely to live in urban areas, have better access to health care, and have more media exposure. Furthermore, because higher education correlates with higher income, have the ability to buy post abortion therapy drug in the pharmacy and have better communication with the health care providers. Also, the women who are living solely & with 2 to 4 people are 53% & 89% less likely satisfied on quality of abortion service than women living together with more than 5 family members. Hence, a women living alone and small family member may have limited information about the quality of safe abortion services and other health care accessibilities whereas a woman living with multiple family members may be able to share financial concerns and receive a suitable location for safe abortions.

Similarly, women who want to have a baby were 56% less likely to be satisfied on the quality of safe abortion service than their counterparts. Lower satisfaction could be due to a woman’s desire for a child and family pressure to have children; these women could also be unaware of the counseling services and pain management therapy drugs available through health care. Furthermore, because unwanted abortion can lead to depression and anxiety both before and after the procedure, women who want to end their pregnancy should have access to accurate information, compassionate counseling, and dependable post-abortion treatment.

Moreover, the post-abortion contraceptive user women are 1.70 times more satisfied than none user women’s consistent with a study conducted in Southern Ethiopia. As a result, the fact that women who had previously had an induced abortion were more likely to use post-abortion contraception, especially long-acting methods, is encouraging. Furthermore, abortion was frequently observed in unmarried women, implying that they were unaware of the availability and accessibility of contraceptives prior to abortion, particularly in rural areas. Furthermore, even if the abortion is performed on a married woman whose husband or religious father may object to contraception, this is an opportunity to receive counseling and additional information about post-abortion prevention and treatment techniques.

## Limitation of the study

The reliability and validity of the satisfaction measures in this study are strong. Second, it was carried out over a large geographic area with a large population. This study has limitations as well; data was collected at a single point in time, so it has the typical limitations associated with cross-sectional research designs. The fact that no response rates were included in the study can also be seen as a limitation. However, because the missing participants were fairly uniform in terms of key sociodemographic variables, their absence may not have had a significant impact on the study’s findings.

## Conclusion

Only one-fourth of the women in this study were satisfied with the safe abortion service’s efficiency. Family size, women’s educational status, maternity status, and post-abortion contraception use were predictors of women’s satisfaction with the quality of abortion. The government, particularly government health institutions, should improve abortion and family planning training and mentoring programs. Furthermore, Ethiopia’s government should focus on addressing contraception needs in order to prevent women from having multiple abortions.

## Supplementary Information

**Additional file 1.**

## Data Availability

The data set analyzed for this study is not publicly available due to restrictions in the IRB consent but may be available from the corresponding author based on reasonable request.

## Abbreviations

AIDS: Acquired immunodeficiency syndrome
HIV: Human immunodeficiency virus
NGO: Non-governmental organization
WHO: World health organization
