# Supplementary material for: Prevalence and determinants of women’s satisfaction on the quality of safe abortion service in Northwest Ethiopia
Source: Arch Public Health. 2022 May 26;80:146. doi: 10.1186/s13690-022-00897-0 (PMC9134683; doi:10.1186/s13690-022-00897-0)
Supplement: Supplementary file 1 — Additional file 1. [file 13690_2022_897_MOESM1_ESM.docx]

**Author’s response to a review**

Prevalence and determinants of women's satisfaction on the quality of safe abortion service in Northwest Ethiopia

To: Archives of Public Health Editorial Office

**Subject: Submitting a revised version of the manuscript**

**Object:** Manuscript AOPH-D-21-00753: Prevalence and determinants of women's satisfaction on the quality of safe abortion service in Northwest Ethiopia

We would like to thank the reviewers and editor for sharing their view and novel scholarly experiences. The comments are very imperative which we strongly believe in improving the manuscript. The point-by-point responses for each of the comments, questions, and the revised manuscript are provided in the attached documents.

**Reviewer#1**

Thank you for providing me an opportunity to review this manuscript. This is an interesting work to read and recommend some corrections, which if corrected can definitely increase the scope of the paper and its significance in the attempted research question.

The objective of the research work is very novel and has covered an important public health problem.

General Observations * was the private versus public health care service provision disintegrated?

Author response: Yes there is a difference regarding the services provided by the private and public health cares in Ethiopia??

* Women's satisfaction level measure needs to be included in the supplementary file, since it is not clear which dimensions of measuring the satisfication were considered in the study.

Author response: Thank you for the suggestion; I already submit it

* Association is mostly explained in terms of significance, but most of the variables does not signify clear association despite being significant.

Author response: thank you for the suggestion; I correct it

* Whether educated women are more satisfied about the quality of abortion or abortion being a safe practice. In the first case then it doesn't mean the logical conclusion (L 210-211).

Author response: thank you for the suggestion; educated women’s are more satisfied on the service provided by quality of safe abortion practice in the study area.

* Quality of abortion will be mainly determined by institutional practices so those factors have not been taken into consideration. Like abortion taking place at private versus public hospital.

Author response: thank you for the comments; mostly in Ethiopia abortion is performed in private clinics rather than government hospitals because in government hospital the process strong due to the legality issues while in private hospital they take low responsibility, considered as normal way on the health worker side they take low responsibility just it assumes one of the source of business.

* Factors like what according to them is a safe abortion or what constitutes a good abortion may also have been taken into account while examining the satisfication level of abortion service provided, which I have found missing in this study so far.

Author response:

* From a client perspective it would have been also important to consider factors like cost involved, technical competence and facility level factors as the determinants.

Author response: thank for the suggestion; I will try to state in figure 1

* Discussion needs to be revised.

Author response: thank you for the suggestion; I already revised it

Specific observations * L204-205 says patients were happy with services at private hospitals, but they were less happy -for abortion services (L224-225). Where the services different when the question was asked.

Author response: Thank you for the suggestion; some times in the case of abortion service the health care provider have low attention on the clients care and they may not provide well counselling service.

* Satisfaction level needs to be studied by level of health car which may provide the better picture of the problem.

Author response: thank you for the suggestion; we accept the comments, however the level of health care in case of Ethiopia still low and have great problem both in government and private health institutions. Even though I will take a look in the next project for further intervention.

* Health care facilities are key to women's satisfication from receiving any services, these factors were either not complexly taken into account or not considered in the final analysis which differentiates from the objective of the study.

Author response: thank you for the suggestion; I will consider it.

* Why was type of care not differentiated based on the institutional services provided like public, private or other NGO?

Author response: Thank you for the comments; in case of government health institution, abortion have limited services due to legal issues and the health care providers recommend to receive the service in private and NGOs as well as the clients are mostly prefer to deliver in NGOS and private sectors due to privacy issues hence they believe that they may get stigma in the community because abortion is not allowed in the Ethiopian community. In the case of private health institution, they believe that abortion considered as a source of income and they provide each abortion service in secure ways, they take keep not told any friends in the time of abortion while in NGOs they prefer to receive the service due to costs and they believe special treatments and counselling hence most of Ethiopians are live within low economic status.

* L 224-226 if women are less satisfied with private health care services why would they opt for that even when bearing the higher expenses. The question here is not about opting for services, but why private healthcare services fail to optimize the effective health care services. The discussion needs some more evidences as the justification provided is not sufficient

Author response: thank you for the genuine comments; in case of Ethiopia’s private health institution perceive that the patient is the source of individual business due to this perception of the private health institution owners optimize the service of the institution was low.

* Why is limitation of the study left blank?

Author response: thank you for the suggestion; I already add it (see page 12, line 257-263).

* Why is health facility not included as a covariate in the regression table? Regression tables needs some more clarification. Like what was the reference category for both dependent and independent variable, significance level should be clearly stated and also some more covariates needs to be included in the table.

Author response: thank you for the suggestion; I already add it see page, table

* Figure 1 is not clear about what it is depicting.

Author response: thank you for the question; ok we make it clear and understandable way.

* Numbering of figures is not clear every figure is denoted with the same number

Author response: Thank you for the suggestion; I have correct it.

* L25 pleased can be replaced by the other word like satisfied

Author response: Thank you for the suggestion; I correct it (see page 2, line 27).

**Reviewer#2**

Dear authors, I have read your manuscript with great interest. I think the study is worthwhile publishing. It adds up to information about patient satisfaction with a specific kind of health service. The low level of satisfaction is remarkable and should be reported and explained.

However, the manuscript needs improvement on language (many typos), the lay out of the tables and figures and even seems incomplete (see line 155).

Author response: Thank you for the suggestion; I correct it

Also I would like you to deepen the discussion by the use of patient satisfaction theory and clear up some points of the discussion, especially the relationship between the use of post-abortion contraceptives and satisfaction with treatment. It looks like a quality of care theory is used for construction of the questionnaire (which is appropriate), but explicit references to such a theory are missing. So, I would not accept the manuscript in its present form, but ask for a revision. In general. Although the study is restricted to the level of satisfaction (which is information), I would like the authors to reflects on the expectancy-experience-value theory, usually underlying levels of satisfaction in relation to the very low level of satisfaction found in this study (see also Harteloh, 2003. The meaning of quality in health care. A conceptual analysis. Health Care Analysis 2003; 11 (3) 259-267.) Could the valuation of the treatment affect the satisfaction with this treatment? (Or How can you be satisfied with an abortion anyway?) Can the authors distinguish between expectations and experiences towards the treatment in their study material? (See also Linder-Peltz, soc sci med 1982, 6, 577-582 (bases for PS theory). Major issue to be resolved is also why post-abortion contraceptives affect the satisfaction of treatment? I cannot see the connection with the treatment and think the discussion on this is not clear.

Author response: Thank you for strong suggestions; I correct it

To me the relationship seems to be inverse: if women are not satisfied with the abortion (at all), they are likely to use contraceptives in future to prevent unwanted pregnancy. However, this is not related to the treatment as such and seems to underpin the satisfaction value theory.

Author response: Thank you for strong suggestions; sometimes due to family influence they may need child and ignores contraceptive in the time of abortion.

The manuscript needs improvement on the following: 1. Typos: line 99: factors instead of factories! line 111: created instead of creat line 135: a code instead of code line139: what is meant by review of original paper? line 149-152: sentence not clear line 152: what is meant by the maximum number? not clear. line 167: give round numbers. line 174: cases in stead of case line 223: this study? line 248: why? explanation is not so clear. See above. line 255: limitations seem to be missing All tables and figures: plural of woman = women instead of women's, also check text. Table 1: heading of third column is missing? All figures are numbered 1. Please revise numbering. 2. please revise layout tables 1 and 2: two major columns with three subheadings each? 3. explanation of table 3 seems to be missing. Explain: COR? AOR? CI? *? **? 4. references 12, 14 etc. seem to mention only the initials of authors? Please mention author. 5. about method: What was the size of the source population? How many women addressed refused participation in this study? There are 618 respondents and 628 institutions (line 150): does it mean 1 respondent per institution? Please explain. 6. line 163-165: amount seems to add up to more than 100%. Please explain. 7. please give round percentages in text (e.g. line 183 29.36 is meaningless and not in accordance with 24% in line 184, etc.). 8. line 183. is there also overlap between financial reasons and social pressure? Or what percentage answers yes to both questions? 9. with regard to results: does the level of satisfaction differ by method of abortion (medication or manual/vacuum aspiration)

Author’s response: thank you for comments; we correct the documents accordingly.
